# Clinical application value of preconception and prenatal carrier screening in Yinchuan

**DOI:** 10.3389/fgene.2026.1832768

**Published:** 2026-07-31

**Authors:** Hua Han, Xiaoyan Zhu, Lifen Chai, Yingchun Ha, Fuying Zhao, Lihua Pan

**Affiliations:** 1 Yinchuan Maternity and Child Healthcare Hospital, Prenatal Diagnosis Center, Yinchuan, China; 2 General Hospital of Ningxia Medical University, Prenatal Diagnosis Center, Yinchuan, China

**Keywords:** expanded carrier screening, next-generation sequencing, preconception screening, prenatal screening, recessive genetic disorders

## Abstract

**Objective:**

To explore the clinical application value of Expanded Carrier Screening (ECS) in preconception and prenatal populations in Yinchuan.

**Methods:**

A total of 1,319 participants underwent ECS, including 1,063 females receiving preconception or prenatal screening and 256 males. Among them, 157 couples completed full paired ECS testing, while the remaining participants only received single-individual testing.

**Results:**

332 individuals were identified as carriers of single-gene diseases, with a total carrier rate of 25.17%. The GJB2 gene had the highest mutation frequency, with a carrier rate of 8.87%, followed by PAH, SLC26A4, and ATP7B. A total of 5 high-risk couples (5/157, 3.18%) were identified. After genetic counseling, 2 couples underwent prenatal amniotic fluid diagnosis: one case was diagnosed with Duchenne/Becker muscular dystrophy and opted for induced abortion, while one couple was a carrier of the GJB2 gene, and the newborn passed the hearing screening test. A pregnant woman had a spontaneous abortion of unknown cause, and a carrier of the GJB2 gene, declined prenatal diagnosis and has not yet delivered. The last couple are carriers of GJB2 gene, and the woman is not yet pregnant, comprehensive genetic counseling has been provided regarding transmission risks and available reproductive options.

**Conclusion:**

ECS is a useful tool for identifying high-risk couples in preconception and prenatal settings and for informing genetic counseling and reproductive planning.

## 1 Introduction

Monogenic disorders represent a major cause of congenital anomalies in newborns. Epidemiological studies indicate that monogenic disorders affect approximately 1% of live births, and between 36.7% and 57.0% of critically-ill infants are diagnosed with such conditions, which severely impact the quality of the birth population and the wellbeing of affected families (Zhu et al., 2022). The lifetime cost of raising a child with a monogenic disorder is extremely high. According to estimates from 2010, these children incur annual expenses of up to $39 million (Mattar et al., 2023), placing a tremendous economic burden on both society and affected families. Expanded carrier screening (ECS) is an important public health strategy for the prevention of genetic diseases. Particularly during the preconception and prenatal stages, it identifies potential carriers of pathogenic variants, providing families with a basis for early intervention and informed reproductive decision-making. However, population-specific carrier rates and mutation spectra in Yinchuan, Ningxia, remain poorly understood. This study aims to investigate the effectiveness of ECS in identifying high-risk individuals during the preconception or prenatal stages in Yinchuan, providing a scientific basis for the prevention and management of monogenic genetic disorders, and supporting the development and implementation of public health strategies in this area.

## 2 Materials and methods

### 2.1 General information

This retrospective study enrolled participants who received prenatal care at Yinchuan Maternity and Child Healthcare Hospital from May 2023 to February 2025. All participants provided written informed consent after being fully informed of the purpose and significance of ECS and voluntarily underwent ECS testing (genes and diseases listed in Table 1). Two screening strategies were applied in this cohort: sequential testing and concurrent testing. For sequential testing, only female participants initially received ECS; male partners were tested subsequently if females carried pathogenic or likely pathogenic variants of autosomal recessive or X-linked disorders. For concurrent testing, both partners of a couple completed ECS at the same consultation visit. The study was reviewed and approved by the Ethics Committee of Yinchuan Maternity and Child Healthcare Hospital (2022-76 and 2024-50).

**TABLE 1:** Genes and diseases list of the expanded carrier screening panel.

Number	Genes	Locus and detection description	Diseases
1	*SMN1*	Exon 7 deletion	Spinal muscular atrophy
2	*DMD (Female only)*	Full-length detection; report pathogenic (P)/likely pathogenic (LP) variants and exon-level deletions/duplications	Duchenne/Becker muscular dystrophy/Dilated cardiomyopathy type 3B
3	*SLC26A4*	Full-length detection; report pathogenic (P)/likely pathogenic (LP) variants	Autosomal recessive deafness type 4 with enlarged vestibular aqueduct
4	*GJB2*	Full-length detection; report pathogenic (P)/likely pathogenic (LP) variants	Autosomal recessive deafness type 1A
5	*HBA1/HBA2*	Full-length detection; report pathogenic (P)/likely pathogenic (LP) variants and five common deletions: -α^3^·^7^, -α^4^·^2^, --SEA, -FIL, -Thai	Alpha-thalassemia
6	*HBB*	Full-length detection; report pathogenic (P)/likely pathogenic (LP) variants	Beta-thalassemia/Sickle cell anemia
7	*PAH*	Full-length detection; report pathogenic (P)/likely pathogenic (LP) variants	Phenylalanine hydroxylase deficiency
8	*ATP7B*	Full-length detection; report pathogenic (P)/likely pathogenic (LP) variants	Wilson disease
9	*MMACHC*	Full-length detection; report pathogenic (P)/likely pathogenic (LP) variants	Methylmalonic aciduria with homocystinuria, cblC type
10	*GAA*	Full-length detection; report pathogenic (P)/likely pathogenic (LP) variants	Glycogen storage disease type 2
11	*GALT*	Full-length detection; report pathogenic (P)/likely pathogenic (LP) variants	Galactosemia type I
12	*PKHD1*	Full-length detection; report pathogenic (P)/likely pathogenic (LP) variants	Polycystic kidney disease type 4 with or without polycystic liver disease

#### 2.1.1 Inclusion criteria

We included couples in the preconception period or early pregnancy. Early pregnancy was defined as a gestational age of less than 14 weeks. In this study, screening was generally performed before 16^+6^ gestational weeks. All enrolled participants had no family history of monogenic disorders and presented normal phenotypes.

#### 2.1.2 Exclusion criteria

The exclusion criteria were as follows:

1. Participants with a suspected family history of genetic disorders or those suspected of having a genetic disorder themselves;
2. Participants who had undergone transplantation, stem cell therapy, allogeneic blood transfusion within the past year, or cellular immunotherapy involving exogenous DNA within the past 4 weeks;
3. Individuals who did not consent to participate in the study.

### 2.2 Testing methods and analysis

A 5 mL peripheral blood sample was collected from each participant and sent to Zhejiang Biosan Biochemical Technologies Co., Ltd. for ECS testing. Genomic DNA was extracted from whole blood. DNA libraries were constructed using a commercial next-generation sequencing library preparation kit (Biosan Tech), and purified libraries were quantified with a library quantification kit (Roche) following the manufacturer’s protocols. A custom carrier screening capture probe kit (Biosan Tech) was applied for target enrichment; the probe set covered all exons of target genes and reported pathogenic (P)/likely pathogenic (LP) variants located in intronic regions. This assay was designed to detect single nucleotide variants (SNVs), insertions and deletions within 10 base pairs, as well as partial deletions and duplications. Target region capture combined with high-throughput sequencing was performed on the MGI-DNB-T7 platform. Raw sequencing reads were aligned to the NCBI human reference genome (hg19/GRCh37). Sequencing data were analyzed bioinformatically, and variant interpretation was conducted according to the relevant guidelines of the American College of Medical Genetics and Genomics (ACMG) (Richards et al., 2015). Variants classified as pathogenic (P) or likely pathogenic (LP) were identified and reported. All IGV images and detection figures presented in this study were generated based on next-generation sequencing results and multiplex ligation-dependent probe amplification (MLPA). Testing for X-linked inheritance patterns was limited to female participants. The final analysis determined the carrier status of pathogenic variants. ds and Result Analysis.

The diseases included in the ECS panel were strictly selected based on the following standardized criteria: (1) a known carrier frequency of 1/100 or higher in the general population; (2) definite and clear clinical phenotypes; (3) severe adverse impacts on patients’ quality of life; (4) potential cognitive or physical impairment caused by pathogenic variants; (5) requirement of long-term medical treatment or surgical intervention; (6) early-onset characteristics during infancy or childhood; (7) the possibility of optimizing delivery management and improving neonatal prognosis, as well as guiding parents to master professional postnatal nursing knowledge.

Considering that the carrier frequencies of certain genes in the Chinese population have not been fully clarified, the disease and gene list of this screening panel was compiled based on four reliable sources: (1) published literature focusing on high-frequency variants in the general population and common pathogenic variants in Chinese, East Asian and Southeast Asian populations; (2) high-frequency loss-of-function (LOF) variants of East Asian and Southeast Asian ethnic groups recorded in the Genome Aggregation Database (gnomAD); (3) authoritative professional genetic databases, including the Human Gene Mutation Database (HGMD), Clinical Variation Database (ClinVar) and Leiden Open Variation Database (LOVD); (4) local population genetic database. The detailed list of detected genes in this study is shown in Table 1.

### 2.3 Clinical counseling

1. When one partner is identified as a carrier of a pathogenic variant associated with an autosomal recessive disorder, the other partner should undergo screening to determine whether both carry pathogenic variants in the same gene.
2. If both partners carry pathogenic (P) or likely pathogenic (LP) variants in the same gene, or if the female carries a P or LP variant related to an X-linked disorder, the couple is classified as a high-risk couple. It is recommended that they receive detailed risk assessment and reproductive counseling. Based on ethical considerations, they may be advised to pursue preimplantation genetic testing for monogenic disorders (PGT-M) to assist conception and/or undergo prenatal diagnosis after conception to provide informed reproductive choices for couples.
3. If the couple does not carry pathogenic variants in the same gene, or if the female does not carry a pathogenic variant associated with an X-linked disorder, participants should be informed that, due to technical limitations of the testing, the possibility of having a child affected by a genetic disorder cannot be completely ruled out.

### 2.4 Statistical analysis

Descriptive data analysis was performed with the software, SPSS 26.0.

## 3 Results

### 3.1 Demographic characteristics

A total of 1,319 participants were included in this study, consisting of 1,063 females and 256 males. Among the female participants, 227 (26.06%) received preconception screening. For females undergoing prenatal testing, 610 (57.38%) were at ≤16 gestational weeks, 135 (12.70%) at 16–17 gestational weeks, and 41 (3.86%) at 18–19 gestational weeks. The mean sampling gestational age was 9.87 weeks.

### 3.2 Carrier status

Among the 1,319 subjects, 332 were carriers of single-gene diseases, with an overall carrier rate of 25.17%. Of these, 295 (88.86%) carried one gene variant, 36 (10.84%) carried two gene variants, and 1 (0.30%) carried three gene variants. There were 72 male carriers (28.14%, 72/256) and 260 female carriers (24.46%, 260/1,063). There was no statistically significant difference in carrier rates between genders (P = 0.225).

### 3.3 Gene mutation analysis

This study detected P or LP variants in 12 genes (Table 2). The GJB2 gene variant was the most common, with 117 carriers detected, accounting for 8.87% of the total subjects. This was followed by the PAH and SLC26A4 genes, with carrier rates of 4.93% (56/1,319) and 3.56% (47/1,319), respectively. The carrier rates for ATP7B, SMN1, α-globin genes (HBA1/HBA2), MMACHC, GAA, PKHD1, GALT, HBB, and DMD were 2.50%, 1.90%, 1.90%, 1.36%, 0.91%, 0.76%, 0.68%, 0.53%, and 0.08%, respectively.

**TABLE 2:** ECS pathogenic gene information.

Genes	Related disorders	Mode of inheritance	Carrier rate
*GJB2*	Autosomal recessive nonsyndromic hearing loss 1A	AR,DD	117 (8.87%)
*PAH*	Phenylketonuria	AR	65 (4.93%)
*SLC26A4*	Autosomal recessive deafness with vestibular aqueduct dilation type 4	AR	47 (3.56%)
*ATP7B*	Wilson’s disease	AR	33 (2.50%)
*SMN1*	Spinal muscular atrophy	AR	25 (1.90%)
*HBA1/HBA2*	α-Thalassemia	AR	25 (1.90%)
*MMACHC*	Methylmalonic aciduria combined with homocystinuria (cbl C type)	AR	18 (1.36%)
*GAA*	Glycogen storage disease type 2	AR	12 (0.91%)
*PKHD1*	Polycystic kidney type 4 with or without polycystic liver	AR	10 (0.76%)
*GALT*	Galactosemia type 1	AR	9 (0.68%)
*HBB*	β-thalassemia	AR	7 (0.53%)
*DMD*	Duchenne/Becker muscular dystrophy	XLR	1 (0.08%)

AR, autosomal recessive. Carrier rate is calculated as the number of carriers divided by all 1,319 enrolled subjects, based on individual count rather than allele count.

A total of 371 variants were detected across 12 genes, encompassing 120 different types of variants. Nine variants, accounting for 54.45% (202/371) of all variants, were detected ≥5 times (Table 3). The most common variant was the c.109G>A (p.V37I) variant in the GJB2 gene, detected 81 times with an allele frequency of 6.14%. This was followed by the c.235delC (p.L79Cfs^★^3) variant in the GJB2 gene, detected 27 times (2.05%).

**TABLE 3:** Common gene mutations in ECS.

Genes	Mutations	Detection count	Carrier rate
*GJB2*	c.109G>A(p.V37I)	81	6.14%
*GJB2*	c.235delC (p.L79Cfs^★^3)	27	2.05%
*SMN1*	exon7del(/)	25	1.90%
*HBA1/HBA2*	α3.7 heterozygous variation	22	1.67%
*SLC26A4*	c.919-2A>G(/)	21	1.59%
*PAH*	c.721C>T(p.R241C)	8	0.61%
*ATP7B*	c.2333G>T(p.R778L)	7	0.53%
*ATP7B*	c.3316G>A(p.V1106I)	6	0.45%
*PAH*	c.728G>A(p.R243Q)	5	0.38%

The symbol “(/)” denotes heterozygous variants and has been removed for unified presentation.

Exon 7 deletions were detected 25 times (1.09%) in the SMN1 gene, the -α^3.7^ variant in HBA1/HBA2 was detected 22 times (0.67%), and the c.919-2A>G variant in the SLC26A4 gene was detected 21 times (1.67%). The PAH gene c.721C>T (p.R241C), the ATP7B gene c.2333G>T (p.R778L) and c.3316G>A (p.V1106I), and the PAH gene c.728G>A (p.R243Q) were detected 8 times (0.61%), 7 times (0.53%), 6 times (0.45%), and 5 times (0.38%), respectively.

### 3.4 Testing results and clinical decisions for high-risk couples

**FIGURE 1:**
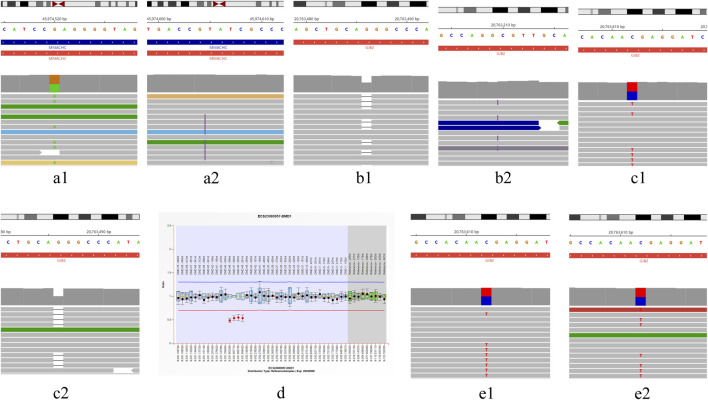
Results of high-risk couple variants a1: c.482G>A(p.R161Q) in MMACHC; a2: c.567dup(p.I190Yfs^★^13) in MMACHC; b1: c.235delC(p.L79Cfs*3) in GJB2; b2: c.508_511dup(p.A171Efs*40) in GJB2; c1: c.109G>A(p.V37I) in GJB2; c2: c.235delC(p.L79Cfs*3) in GJB2; d: exon45_48del(/) in the DMD gene; e1: c.109G>A(p.V37I) in GJB2; e2: c.109G>A(p.V37I) in GJB2.

In this study, 157 couples underwent ECS testing, and 5 couples (3.18%) were identified as high-risk (Table 4). Genetic testing results are shown in Figure 1. Couple #1 both had pathogenic variants in the MMACHC gene, but experienced unexplained miscarriage during pregnancy. Copy number variation testing of the miscarriage tissue was normal. The couple refused whole-exome sequencing, and the possibility of a single-gene disease could not be ruled out. They were advised to consider PGT-M assisted reproduction or prenatal diagnosis after conception to rule out the MMACHC gene risk for future pregnancies. Two high-risk couples both carried pathogenic variants in the GJB2 gene. In one couple (#2), the woman underwent genetic counseling and prenatal amniocentesis, and the fetus was found to be a GJB2 carrier (consistent with the mother). After vaginal delivery, the fetus passed hearing screening, and the couple was advised to consider PGT-M assisted reproduction or prenatal diagnosis after conception if they planned to conceive again. The other couple (#3) refused prenatal diagnosis; the fetus has not yet been delivered, and the clinical outcome is unknown. The female partner of the couple #4 carries the pathogenic mutation of the DMD gene and has a family history of it. Prenatal amniocentesis revealed DMD in the fetus (identical to the mother). Labor was induced, and the couple was advised to consider PGT-M assisted reproduction for future pregnancies. The female partner of couple #5 is not yet pregnant. Genetic counseling has informed her of the risks and fertility strategies.

**TABLE 4:** Outcomes for high-risk couples.

Number	Gender	Age	Gestational age	Genes	Related disorders	Gene mutation	Genotype	Variation classification
1	Female	30	​	*MMACHC*	Methylmalonic aciduria combined with homocystinuria (cbl C type)	c.482G>A (p.R161Q)	Heterozygous	P
	Male	32	-			c.567dup (p.I190Yfs^★^13)	Heterozygous	P
2	Female	26	13w+2	*GJB2*	Autosomal recessive nonsyndromic hearing loss 1A	c.235delC (p.L79Cfs*3)	Heterozygous	P
	Male	27	-			c.508_511dup (p.A171Efs^★^40)	Heterozygous	P
3	Female	28	15w+4	*GJB2*	Autosomal recessive deafness type 1A	c.235delC (p.L79Cfs*3)	Heterozygous	P
	Male	37	-			c.109G>A (p.V37I)	Heterozygous	P
4	Female	22	13w+0	*DMD*	Duchenne/Becker muscular dystrophy	exon45_exon48del	Heterozygous	P
5	Female	28	Pre-pregnancy	*GJB2*	Autosomal recessive nonsyndromic hearing loss 1A	c.109G>A (p.V37I)	Heterozygous	P
	Male	31	​			c.109G>A (p.V37I)	Heterozygous	P

“Number” represents the sequential serial number of high-risk couples, with no additional clinical implication.

## 4 Discussion

This retrospective study evaluated the clinical application value of ECS covering 12 common recessive monogenic disorders among 1,319 preconception or prenatal subjects in Yinchuan. The results showed that the overall carrier rate of pathogenic and likely pathogenic variants reached 25.17%, with GJB2, PAH and SLC26A4 ranking as the top three genes with the highest carrier frequencies. Among 157 couples who completed paired testing, 5 high-risk couples (3.18%) were identified and provided with targeted genetic counseling and reproductive guidance. These findings preliminarily verify that ECS can effectively identify hereditary disease risks in the local population and provide reliable evidence to support clinical reproductive decision-making.

The panel design of ECS needs to balance clinical benefit and economic feasibility. Currently, there is no global consensus on the optimal number of genes for ECS panels, with reported panel sizes ranging from 41 to 1,792 genes (Chokoshvili et al., 2018). Previous large-scale domestic studies have confirmed that targeted screening focusing on high-frequency pathogenic genes can achieve high coverage of high-risk couples while effectively controlling testing costs (Sun et al., 2024). Given the limited medical resources and actual economic conditions in Yinchuan, this study selected 12 genes with well-documented high carrier frequencies to construct the screening panel, so as to improve the cost-effectiveness of testing and better adapt to large-scale population screening in the local area.

The overall carrier rate in this study reached 25.17% based on a 12-gene panel. This rate higher than the carrier rate of 1.73% observed in a study involving 18818 women screened for spinal muscular atrophy (SMA), which excluded 10 carriers with a history of bearing children affected by SMA (Sun et al., 2024), and also higher than the 5.7% positive rate reported by Mohan et al. in a noninvasive prenatal screening panel targeting 25 clinically significant monogenic diseases (Mohan et al., 2022). However, it is lower than that reported by Tan et al., who performed a 108-gene panel screening with a carrier rate of 31.7% (Tan et al., 2024), and lower than the 47.8% carrier rate reported by Chau et al. in a study of 1,543 individuals from southern China screened for 315 genes (Chau et al., 2022). The discrepancies across studies are mainly attributed to differences in gene panel size, study population characteristics, screening strategies, detection scopes and sequencing platforms.

In terms of variant spectrum, 120 distinct variants were detected in this study, and the five most common variants (detected ≥21 times) accounted for 47.44% (176/371) of all mutations, indicating a high degree of genetic heterogeneity and regional specificity. Notably, the GJB2 c.109G>A variant showed the highest carrier frequency, differing from previous reports identifying c.235delC as the most common variant among northern Chinese populations (Bian et al., 2022), where 235delC is also recognized as the hotspot mutation in GJB2 among patients with nonsyndromic hearing impairment (NSHI) (Yu et al., 2007). Additionally, the carrier rate of the–α^3.7^ deletion was much higher than that of – –^SEA^ (1.67% vs. 0.15%), which also contrasts with earlier reports from other regions in China (Yang et al., 2019). These findings suggest that the Ningxia region may have unique mutational characteristics influenced by geographic or population-specific factors.

The combined carrier frequencies of GJB2 and SLC26A4 variants were significantly higher than the national averages (8.87% vs. 2.53%; 3.56% vs. 2.05%, respectively) (Zhang et al., 2023). This may be closely related to the distinctive ethnic genetic makeup of Yinchuan, where the Hui population constitutes a large share of local residents. Previous local neonatal screening data have demonstrated population-specific differences in deafness gene variant spectra between Han and Hui subgroups in this area, with a higher proportion of deafness gene carriers identified among Hui individuals (Gu et al., 2020), which collectively elevates the overall carrier prevalence within the regional preconception population. In addition, phenylketonuria (PKU) has a relatively high incidence in Ningxia, ranking second nationwide (Xiang et al., 2019; Yu et al., 2023). Studies in Yinchuan reported hotspot mutations in the PAH gene as p. R252 W and p. R261Q, accounting for 17.9% and 14.3% of the mutation types, respectively (Yu et al., 2023). However, in this screening, the p. R252 W mutation was found, and the p. R261Q was only detected once. This may be due to the relatively small sample size in this study, which affected the data analysis results.

ECS aims to broadly screen healthy individuals to assess their likelihood of having autosomal recessive (AR) and X-linked (XL) diseases (Sagaser et al., 2023). Screening timing is critical for ECS implementation, as sufficient time must be reserved for subsequent genetic counseling and prenatal diagnosis. It is generally recommended to complete ECS sampling before 15 gestational weeks. In this study, however, 57.89% of pregnant participants underwent screening after 15 weeks, and 16.54% were tested after 18 weeks. Although no adverse clinical outcomes occurred in these cases, relatively late sampling still increases the risk of delayed reproductive decision-making. This indicates that local health education on ECS needs to be strengthened to optimize screening timing and ensure the timeliness of prenatal diagnosis.

In this study, only 21.92% of male partners of female carriers completed sequential testing, which was far lower than the 77% reported in previous reports from abroad (77%) (Simone et al., 2021). In the sequential screening protocol of this study, male partners were only recalled for testing when the female partner was identified as a carrier of pathogenic/likely pathogenic variants; males who chose simultaneous screening were not included in the sequential recall statistics. In addition, the low recall rate is also affected by multiple factors such as adequacy of health education, public awareness, psychological acceptance and communication costs. Among the 157 couples who completed paired testing, 5 (3.18%) were confirmed as high-risk couples. This proportion was lower than the 5.24% (874/16669) in the domestic big data (Sun et al., 2024), but slightly higher than the 2.43% (255/10476) screening data of 10476 couples in five southern provinces in 2019 for 11 single-gene diseases (Zhao et al., 2019). It can be seen that the detection of high-risk couples is closely related to the types and number of diseases included, in addition to regional differences, ethnicity, target population, and sample size.

This study has several limitations. First, it is a single-center retrospective design with a relatively small sample. Second, only 12 genes were included, which may underdiagnose some pathogenic variants. Third, the male partner screening rate was low (21.92%). Future multi-center, large-sample studies with broader gene panels are needed.

## 5 Conclusion

The application of ECS in Yinchuan demonstrates significant potential for identifying high-risk couples and supporting genetic counseling, clinical decision-making, and reproductive guidance. However, the limited testing coverage in this study may have resulted in the omission of pathogenic variants in untested genes. These limitations highlight the need for further refinement and optimization in future research and clinical practice. In practical implementation, continuous improvement of testing strategies and workflows is essential to enhance screening efficiency, reduce the incidence of genetic diseases, and provide stronger evidence to support the development and execution of public health policies.

## Data Availability

The original contributions presented in the study are included in the article/supplementary material, further inquiries can be directed to the corresponding authors.
